# Beta-Blockers and Cancer: Where Are We?

**DOI:** 10.3390/ph13060105

**Published:** 2020-05-26

**Authors:** Rita Peixoto, Maria de Lourdes Pereira, Miguel Oliveira

**Affiliations:** 1 Department of Biology, University of Aveiro, 3810-193 Aveiro, Portugal; ritajesuspeixoto@ua.pt; 2 CICECO–Aveiro Institute of Materials, Department of Medical Sciences, University of Aveiro, 3810-193 Aveiro, Portugal; 3 Centre for Environmental and Marine Studies (CESAM), Department of Biology, University of Aveiro, 3810-193 Aveiro, Portugal

**Keywords:** beta-blockers, therapeutic application, cancer

## Abstract

Cancer is one of the leading causes of death worldwide. After diagnosis, cancer treatment may involve radiotherapy, chemotherapy, and surgery. Several of the approaches used to treat cancer also attack normal cells and, thus, there is the need for more effective treatments that decrease the toxicity to normal cells and increase the success rates of treatment. The use of beta-blockers in cancer has been studied for their antagonist action on the adrenergic system through inhibition of beta-adrenergic receptors. Besides regulating processes such as blood pressure, heart rate, and airway strength or reactivity, beta-blockers block mechanisms that trigger tumorigenesis, angiogenesis, and tumor metastasis. This study presents a literature review of the available studies addressing cancer treatments and beta-blockers. Overall, data suggest that propranolol may be used as a complement for the treatment of several types of cancer due to its ability to improve cancer outcomes by decreasing cancer cell proliferation rates. Nonetheless, additional in vitro studies should be performed to fully understand the protective role of BBs in cancer patients.

## 1. Introduction

**Figure 1:**
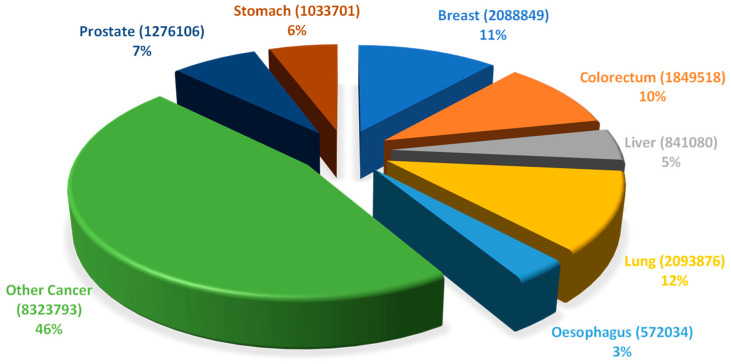
Distribution of the estimated number of worldwide cancer cases in 2018 (18,078,957) per type of cancer. Data includes all types of cancers, all ages and both sexes (Adapted from Global Cancer Observatory—World Health Organization [2]).

Cancer, a group of abnormalities characterized by unmeasured growth of cells leading to the development of tumors, is a global public health problem, at the top of the leading causes of death in wealthy countries (CDC, 2020). The global cancer burden is significant and increasing. According to the National Center for Health Statistics of the United States of America, the most commonly diagnosed cancers in men are prostate, lung, and colorectal cancer, whereas, in women breast, lung, and colorectal cancer are the most common [1]. Figure 1 presents the distribution of the estimated cancer cases worldwide (18,078,957), per types of cancers.

It is estimated that each year 9.6 million people die from cancer, and that a quarter of those deaths are related to lung cancer. The five-year survival rate for patients diagnosed with cancers is lower for pancreas (9%), increasing for liver (18%), esophagus (19%), and lung (19%) cancers [3]. During their lifetime, one in five men and one in six women worldwide will develop a type of cancer [4]. Once diagnosed, the treatment of patients may involve different approaches that include radiotherapy, chemotherapy, and surgery. Primary prevention, screening and early diagnosis, multimodal treatment and survival and palliative care are the spectrum of cancer control interventions. There are significant differences in terms of cost of treatment, with estimates of 25,000 Canadian dollars for melanoma, thyroid, and testicular cancers and 60,000 Canadian dollars for leukemia. Lifetime treatment costs may range from 55,000 Canadian dollars for lung and liver cancers to over 110,000 Canadian dollars for leukemia, lymphoma and breast cancer [3].

## 2. Beta-Blockers

The expression of specific receptors (proteins able to bind ligands (e.g., catecholamines) and transducing extracellular signals across the plasma membrane) and the activation of intracellular signaling pathways is a key process of cells. These specificities enable cells to interact and adapt to the surrounding environment. Beta-blockers (BBs) are commonly considered cardioprotective drugs used in various diseases (e.g., hypertension or coronary artery disease) due to their antagonist action on the adrenergic system through inhibition of beta-adrenergic receptors [5,6,7,8,9]. BBs have been considered for cancer treatment due to their antagonist action on receptors associated with mechanisms that trigger tumorigenesis, angiogenesis, and tumor metastasis, which may allow the decrease of the enormous costs of cancer treatments, as well as short survival rates [10].

BBs were first discovered in 1906 by Sir Henry Hallett Dale, awarded with a Nobel prize for his discovery. However, it was only in 1948 that Raymond Perry Ahlquist observed that adrenergic receptors could be divided into two types (alfa- and beta-receptors). In 1967, Alonzo M. Lands observed that, depending on the tissue, BBs could act by two different pathways, culminating in the differentiation of beta-adrenergic receptors into two subtypes: beta-1 and beta-2 subtypes. Meanwhile, it was discovered that some BBs may act on both pathways, acting on both receptor subtypes. An example of this type of drugs is propranolol, the prototype of the first invented BBs and the one with the most collected experience and clinical indications [11].

The adrenergic receptors, members of the superfamily of cell surface receptors that carry out signaling via coupling to guanine nucleotide binding proteins (G-proteins) can be divided into 2 types: alfa-receptors (associated with “excitatory” functions such as vasoconstriction) and beta-receptors (associated with “inhibitory” functions like vasodilatation and excitatory effects in the myocardium) [12,13,14,15,16,17]. Beta-receptors are divided into three subtypes: beta-1-receptors (commonly associated with the heart), beta-2-receptors (responsible for vascular and airway relaxation), and beta-3-receptors (present in the cells of brown adipose tissue from rats) [18,19]. In this perspective, an agent able to inhibit the response of the adrenergic receptors is an adrenergic antagonist, whereas, a molecule stimulator of response (e.g., catecholamines) is an adrenergic agonist [17]. Thus, based on the affinity to the beta-subtype receptors, BBs can be considered as “beta-1 selective” or “cardioselective” (as the beta-1 subtype is the predominant one in the heart) when exhibiting a higher affinity for beta-1 subtype than for beta-2 (e.g., atenolol, celiprolol, metoprolol, bisoprolol, and nebivolol) or “nonselective BBs” if acting on both beta-1 and beta-2 receptors (e.g., propranolol, sotalol, carvedilol, labetalol, and timolol) [20,21]. Some selective or nonselective BBs are also antagonists of the alfa-1 receptors (carvedilol and labetalol) and alfa-2 receptors (celiprolol) and have the capacity of increasing nitric oxide release (nebivolol and carvedilol), causing a vasodilatory activity [11,22,23,24,25].

The use of this group of molecules of different pharmacokinetic and pharmacodynamic properties has been considered in the treatment of different pathologies like hypertension, cancer, and migraine, suggesting a protective effect that may span far beyond the cardiovascular system [26,27]. The known effects of BBs are diverse. BBs regulate, among other functions, processes like blood pressure, heart rate and airway strength or reactivity [17]. These pharmaceuticals are used worldwide, and its consumption has been increasing, over time, in older patients as BBs are considered to play a protective role in the cardiac muscle. Furthermore, it is believed that patients with hypertension have increased survival rates when taking BBs [28]. However, recent meta-analyses (that do not take age into account) state that BBs are unsuitable for the treatment of hypertension as first-line therapy [29]. BBs have been associated with lower mortality rates in the 5 years following an episode of myocardial infarction in patients with stable coronary heart disease [30]. However, in patients with myocardial infarction without heart failure, BBs showed no beneficial effect when used beyond 1 year after the episode [31]. Studies have, however, suggested side effects of BBs. According to a study performed by the Action to Control Cardiovascular Risk in Diabetes, diabetic patients taking BBs have significantly higher CV disease event rates and increased incidence of hypoglycemia [8,32].

The use of BBs is contraindicated in patients with asthma, reactive airway disease, acute decompensated heart failure with systolic dysfunction, heart block and sick sinus syndrome, even in the therapeutic dose range [33].

## 3. Antineoplastic Agents and Cardiotoxicity

Regardless the type of cancer, cancer treatment aims to control or even terminate the uncontrolled growth of cancer cells [34]. In the last years, new antineoplastic agents have been emerging, presenting advantages in terms of safety, availability, and lower cost when compared to those widely used [35]. These agents play essential roles in triggering, controlling, and modifying cancer cell mechanisms regarding its proliferation, differentiation, and survival [36].

Antineoplastic agents can be divided into nine groups (Table 1): alkylating, alkylating-related, antimetabolite, topoisomerase-1 inhibitors, topoisomerase-2 inhibitors, DNA-intercalating agents, agents that interfere with tubulin, tyrosine-kinases inhibitors, and others [37].

Anthracyclines (e.g., doxorubicin, daunorubicin, epirubicin, and idarubicin) are widely used for the treatment of breast cancer and lymphoma [38]. They act on cancer cells by intercalating between DNA base pairs, disrupting the DNA chain, and they stabilize the topoisomerase 2-alfa complex, preventing the association of disrupted DNA strands and, thus, lead to cell death [39,40]. However, its use has been associated with cardiomyocyte injury, due to the formation of reactive oxygen species (ROS) and inhibition of cardiomyocytes’ topoisomerase 2-beta, and their death associated with double-stranded DNA that lead to apoptosis through the activation of p53 pathway [41,42]. These effects may cause arrhythmias, symptomatic heart failure, and asymptomatic left-ventricular dysfunction which can be quantified through measurement of left-ventricular ejection fraction (LVEF). A decline in LVEF is a strong indicative of heart failure following anthracycline treatment and is more commonly detected in the elderly [41,43]. Arrhythmias may be caused not only by anthracyclines use but also by cisplatin, taxanes, vinca alkaloids, platinum, arsenic, thalidomide, antimetabolites, and interleukin-2 (IL-2) [44]. In a pediatric population, the risk of heart failure may remain high, even decades after treatment with anthracyclines [45].

Cisplatin is an alkylating-related agent that stands between two adjacent guanines followed by an adjacent guanine and adenine, inhibiting DNA replication and transcription and leading to cell death [46]. It is one of the most extensively used drugs for cancer treatment [47,48]. In testicular cancer treatment, a 90% cure rate has been reported in patients using this drug which is also used in the treatment of head and neck, cervical, breast, lung, ovarian, gastric, and bladder cancers [49]. Despite the success of cisplatin in cancer treatment, the reported side effects (e.g., nephrotoxicity, neurotoxicity, and ototoxicity) limit its use [50,51]. Nausea, vomiting, and myelosuppression are other common side effects [52]. Over the last years, cardiotoxic events have been reported for cisplatin treatments, including silent and symptomatic arrhythmias, angina, myocarditis, pericarditis, diastolic disturbances, cardiac ischemia, acute myocardial infarction, thromboembolic events, and chronic heart failure [53]. Considering that treatment with cisplatin is not as strictly monitored as those with other anticancer drugs (e.g., anthracyclines), some cardiotoxic effects may have been undetected or overlooked [49].

Arrhythmia is the most common cardiotoxic effect occurring in treatments with cisplatin (18–32% of patients) [44,54]. Tachycardia, which may be defined as a heart rhythm disorder in which the heart produces electrical signals that lead to faster heart beats (above 100 beats per minute), is the most recurrent type of arrhythmia resulting from treatment with cisplatin. Sinus bradycardia, a clinical term for a heartbeat slower than normal (below 60 beats per minute) [55,56] has also been reported. The cisplatin induction of hypomagnesaemia (Mg serum levels lower than 1.7mg/dL) may be responsible for these side effects as well as tremors and ataxia [49].

In order to decrease the toxicity induced by cancer treatment (chemo- and radiotherapy) to normal cells, new approaches have emerged, such as targeted molecular therapies, aiming at treatments focused on specific targets. New monoclonal antibodies have been approved due to their favorable tolerability profiles and reduced secondary effects. These molecules may also be safely combined with widely used chemotherapeutic agents or radiotherapy [57]. Trastuzumab is a monoclonal antibody used to treat breast cancer. However, its use has been associated with cardiotoxicity, injuring cardiomyocytes, and increasing the risk of heart failure. In a cohort study with 16,456 patients, 4325 of them receiving trastuzumab as treatment for breast cancer, the rate of heart failure incidence was 8.3%, against the 2.7% observed in patients not treated with trastuzumab. The risk was higher in patients treated with anthracyclines and trastuzumab, followed by patients receiving trastuzumab-based chemotherapy and then by patients receiving anthracyclines treatment. As expected, older patients exhibited increased risk of cardiotoxicity. These data are consistent with the fact that older patients presented comorbidities such as hypertension and valve disease. Radiation therapy, however, did not have any influence in the risk of heart failure [58].

The vascular endothelial growth factor (VEGF) receptor inhibitors (antibodies and kinase inhibitors) are anticancer drugs due to their anti-angiogenesis properties. However, cardiac side effects are known to be caused by these agents. They may also cause hypertension, endothelial dysfunction, and increased platelet aggregation. The use of bevacizumab in cancer therapeutics has been related with increased risk of congestive heart failure, but only in breast cancer patients. In a combination therapy including bevacizumab and taxanes, congestive heart failure risk is reported to increase significantly [54].

### 3.1. Cardioprotection during Cancer Therapy

Traditional cardiac risk factors include hypertension, dyslipidemia, smoking, and diabetes mellitus. Additionally, cumulative dose, age, radiotherapy of the left side of the chest, previous exposure to cardiotoxins, and co-administration of anthracyclines and trastuzumab or taxanes and bevacizumab are factors that may increase the risk of cancer treatment-associated cardiotoxicity [54,59].

There are multiple formulations which may mitigate toxic effects of anticancer therapy by altering the drug properties or by protecting cells against its cardiotoxic effects.

The use of liposomal formulations has been considered to improve drug targeting and to reduce toxic effects of doxorubicin, once inside a liposome. As a phospholipid bilayer vesicle, it carries doxorubicin presenting advantages in terms of immunogenicity and toxicity [60]. In a study with 509 metastatic breast cancer patients, who received either liposomal doxorubicin or conventional doxorubicin, investigators assessing LVEF concluded that progression-free survival rate was higher for patients taking liposomal doxorubicin (7.8 months) than for those taking conventional doxorubicin (6.9 months). Although the use of liposomal doxorubicin lead to less secondary effects, both showed similar efficacy [54,61].

Vitamin E has a cardioprotective effect due to its antioxidant properties [54,62]. However, in a human-based study with 13 patients on chemotherapy, no difference in cardiac protection was found between patients treated with and without vitamin E [54,63].

The use of BBs has been proposed to improve relapse-free and overall survival in patients being treated for multiple types of cancer [35]. The induction of endogenous beta agonists (such as catecholamines) is associated with mechanisms that trigger tumorigenesis, angiogenesis, and tumor metastasis. Those mechanisms include the activation of genes associated with metastasis and inflammation, activation of cell proliferation pathways and upregulation of pro-angiogenic factor and VEGF [12]. Considering that mediation via beta-2 receptor seems to be partly responsible for those mechanisms, a non-selective beta-1 and beta-2 receptor antagonist like propranolol should be a more promising potential anti-cancer agent than selective beta-1 receptor antagonists [64]. However, this assumption is dubious since a meta-analysis came to the conclusion that the use of propranolol did not cause any significant difference in cancer specific death rate, overall death rate or relapse-free survival rate between patients taking propranolol and those who did not [65].

Nevertheless, propranolol demonstrates high safety and good tolerability profiles, being recommended for first-line therapy in some CV diseases. Its first indication was for the treatment of angina but soon it was discovered that propranolol was also effective when used for other CV conditions such as hypertension, myocardial infarction, and arrhythmias [66].

### 3.2. BBs and Breast Cancer

The number of successful breast cancer treatments has been increasing in the recent years, partially due to early detection of cancer, better treatment options and multidisciplinary healthcare teams [67]. The use of aromatase inhibitors such as anastrozole, letrozole and exemestane, for the treatment of post-menopausal women, and tamoxifen, for pre-menopausal women, is an available therapy [68]. Secondary effects of these drugs such as impairment of cognitive function—perception, planning, and memory—and psychomotor speed have been reported in some women also experiencing depressive symptoms [69,70,71].

The consumption of BBs during chemotherapy treatment helps improving relapse-free survival in breast cancer women, but not overall as seen in a populational study [72]. Also, breast cancer patients taking BBs before diagnosis presented a significant lower rate of metastasis [35,73,74,75].

A recent review of eight studies revealed that patients receiving treatment for breast cancer with an anthracycline with or without trastuzumab and using BBs presented a significant reduction in heart failure incidence when compared to those not using BBs, which supports the use of these medicines as cardioprotective in patients receiving cardiotoxic treatments [35,76].

A patient with stage III HER2-negative breast cancer type, treated with 1.5 mg/kg/day propranolol for 18 days and with the daily dose reduced over subsequent 7 days, after the treatment period, had the tumor removed surgically [77]. The tissue was collected for analysis to compare pre- and post-treatment tissues. Ki-67 (a pro-proliferative protein) expression decreased with treatment, an indication of tumor proliferation altered by propranolol administration. Bcl-2 (a pro-survival marker) expression decreased after propranolol administration whereas p53 protein (a pro-apoptotic protein) expression increased approximately 2.5-fold [77]. These findings were validated in a study with MDA-MB-231 breast cancer cell line exposed to propranolol (40 µM) and doxorubicin (3 µM) where propranolol reduced the rate of cells arrested in the G_2_/M phase of the cell cycle, showing that cells died or were in the process of dying. These results support the hypothesis that BBs may have some antagonist action on breast cancer cell proliferation. Following 6-h treatment with propranolol, p53 protein expression in MDA-MB-231 cells markedly increased. Thus, propranolol led to increased levels of cleaved initiator caspase 9 and execution caspases 3 and 6, so propranolol may lead to apoptosis of breast cancer cells. The investigators concluded that BBs may decrease breast tumors proliferation. However, additional studies are needed to fully understand the anticancer mechanisms underlying propranolol [77].

The potential of carvedilol to prevent cardiotoxicity during chemotherapy treatments was assessed in a trial test involving 1122 patients aged 18 years or older [78]. The primary endpoint was a decrease of at least 10% in LVEF and the secondary the levels of troponin I (TnI) higher than 0.04 ng/mL. High TnI levels associated with LVEF reduction are indicative of cardiac events [78]. Patients were being treated for HER2-negative breast cancer and the treatment included doxorubicin, cyclophosphamide and paclitaxel. Patients with previous heart failure symptoms, cardiomyopathy, coronary artery disease, mitral aortic disease, and chemo- or radiotherapy history; patients previously treated with angiotensin-converting enzyme inhibitors and BBs; and patients with contraindication to the use of BBs were excluded. Carvedilol and placebo were administered with the beginning dose of 3.125 mg, ascending to 6.25 mg, then 12.5 mg and the maximum dose of 25 mg, every 12 h until completion of chemotherapy. During the follow-up, 27 patients (14%) had a decrease of at least 10% in LVEF, 14 of those were receiving carvedilol, whereas the other 13 were in the placebo group. TnI levels increased in both groups from baseline until the end of the follow-up but its levels were attenuated in the carvedilol-treated group. The investigators concluded that there were no significant changes in LVEF between groups, however, the TnI elevation and further attenuation by carvedilol use suggests that carvedilol may have a protective role against myocardial injuries [79]. This protection is probably due to the antioxidant pharmacological properties and subsequent carvedilol protection against free radicals [78].

Using in vitro cell evaluation, the hypothesis that BBs reduce the proliferation rates of breast tumors when collected in the year prior to diagnosis was assessed. The expression of beta-1, beta-2, and beta-3 adrenergic receptors was measured in breast cancer tissue in contrast to normal breast tissue [80]. Tissues were collected from 404 breast cancer patients. Beta-1 and beta-3 receptors were significantly more expressed in breast cancer tissue than in normal breast tissue, however, there were no differences detected in beta-2 receptors expression. Cells collected from patients in stage I breast cancer who used BBs showed a significant decrease in Ki-67 compared to non-users. The same was observed for stage II breast cancer patients. Moreover, a significant decrease in tumor proliferation was observed in stage I breast cancer patients taking nonselective BBs, but the same was not found in stage II, III, or IV breast cancer patients. To corroborate these results, the investigators administered propranolol, a nonselective BB, to a HER-2 negative breast cancer patient [80]. Ki-67 index was evaluated pre- (through biopsy) and post- (after surgical resection) treatment with propranolol for 25 days (1.5 mg/kg/day). In the post-treatment period, Ki-67 was 23% lower than in the pre-treatment tissue. Therefore, the investigators concluded that propranolol may significantly decrease tumor proliferation. To fully understand the mechanism underlying propranolol, in vitro testing consisting in 24h exposure of SK-BR-3 cells to 18 µM propranolol (EC_50_ for the cell line) was performed [80]. Test results demonstrated decreased phosphorylation of multiple mitogenic activated protein kinases and cyclic adenosine monophosphate (cAMP) responsive element binding protein (CREB), and increased phosphorylation of protein kinase B (PKB), glycogen synthase kinase 3 (GSK3) and p53. These data suggested that propranolol lead to a decrease in cancer cell proliferation and an increase of cell stress [80].

### 3.3. BBs and Ovarian Cancer

Ovarian cancer is the eighth most common cancer in women and its survival rate after 5 years of diagnosis is only approximately 40% [81]. There is, thus, the need to improve ovarian cancer outcomes and to provide better treatment options. In this perspective, BBs could be considered for their action on the adrenergic system [8].

In a population-based study, 9420 patients being treated for ovarian cancer were administered BBs to test if there was an improvement in survival. Patients were given cardioselective BBs or nonselective BBs. During the follow-up (maximum 5 years), 2918 patients (47%) died, with 2051 deaths (70%) due to ovarian cancer. The use of BBs was associated with increased mortality. However, the decreased survival among the users of BBs may be explained by the fact that older patients, consequently having increased prevalence of CV diseases and other comorbidities, were mostly the ones taking BBs [9]. Even though previous studies have shown that BBs have no beneficial protective effect on patients under cancer treatment, the impact of nonselective BBs on survival was higher than cardioselective BBs [9,82,83]. The investigators did not consider confounding covariates such as body mass index, hypertension, CV comorbidities, or other comorbidities. This lack of information was a limitation in this study as CV comorbidities may be an explanation for the increased mortality rates of BBs users [9].

### 3.4. BBs and Pancreatic Cancer

Pancreatic cancer is, currently, the fourth cause of cancer-related death due to the lack of therapeutic strategies. Only a small percentage (5%) of patients with this type of cancer survive for 5 years, the lowest rate of survival among all cancer patients. Adenocarcinoma represents more than 85% of all pancreatic cancers and is the most lethal one [84].

The development of pancreatic cancer is associated with induction of the sympathetic nervous system, which leads to an increase in catecholamines stimulation. Several studies indicate that BBs, particularly the nonselective ones such as propranolol, may inhibit the damage induced by catecholamines stimulation of the adrenoreceptors in pancreatic cancer patients [85,86,87,88,89,90,91].

In a retrospective cohort, the association between BBs exposure and cancer-specific mortality was assessed. Patients recently diagnosed with pancreatic adenocarcinoma (2394) were, during the 4 years of follow-up, given a certain type of BB in the generally prescribed dose for adults. During the follow-up, 91% of patients (2187) died (2054 of pancreatic cancer, 33 of CV disease, and 100 of other causes) and the median survival was 5.1 months for all the patients. Of the patients, 522 were treated with nonselective beta-blocker propranolol or cardioselective BBs metoprolol, atenolol, or bisoprolol and the rest of the patients with a combination of BBs formulas and alpha-1 blockers or other anti-hypertensive agents. BBs use, when compared with nonuse, was associated with an overall reduced cancer-specific mortality rate. Due to poor use of nonselective BB propranolol, authors listed no significant differences between nonselective and cardioselective BBs use. Authors concluded that BBs may inhibit progression of pancreatic adenocarcinoma and may be a complement for current therapies in order to prevent cell damage in pancreatic cancer patients [91]. In this study, other medications used by patients, such as other antihypertensive drugs, antidepressants, anxiolytics, antipsychotics, aspirin, nonsteroidal anti-inflammatories, statins, digoxin, metformin, insulin, and other hypoglycemic agents were considered. Blockers of angiotensin II receptors, serotonin and norepinephrine reuptake inhibitors, diuretics, nonsteroidal anti-inflammatory drugs and antipsychotics were associated with increased rate of cancer-specific mortality, whereas the use of anxiolytics was associated with a statistically significant reduced rate of cancer-specific mortality. Since anxiolytics (such as benzodiazepines) act by reducing norepinephrine release through potentiation of gamma-aminobutyric acid (GABA) at the GABA receptors, it is assumed that their influence in the central nervous system may be related with less damage caused by catecholamines, leading to a decreased rate of cancer-specific mortality, following the reduced rate of cancer-specific mortality seen in those individuals [92,93,94].

### 3.5. BBs and Liver Cancer

Liver cancer accounts for the second deadliest mortality rate caused by malignant cancers in men. Liver cancer develops quickly but it is asymptomatic [95].

Propranolol has been administered to patients with liver cancer and liver cirrhosis as a prevention for esophageal and gastric variceal hemorrhage. Variceal bleeding is one of the major causes of death in cirrhotic patients. In cirrhosis, portal pressure initially increases as a consequence of resistance to blood flow within the liver. This resistance is due mainly to fibrous tissue and regenerative nodules in the hepatic parenchyma. Therefore, propranolol should be able to reduce portal vein pressure, exhibiting an anticancer effect [96].

In a cell-based study (with HepG2 and HepG2.2.15 liver cancer cells and HL-7702 normal human liver cells), it was demonstrated that propranolol (80 µM) inhibits the proliferation of liver cancer cells (HepG2 and HepG2.2.15) but not normal cells (HL-7702). At the same time point, progressive propranolol concentrations lead to a pronounced inhibitory effect of liver cancer cell proliferation. Treatment with 160 µM resulted in shrinkage of cell lines HepG2 and HepG2.2.15 and 320 µM propranolol resulted in shrinkage of all three cell lines. Both beta-1 and beta-2 receptors were expressed on the membrane of the three cell lines, but expression was higher on HepG2 and HepG2.2.15 cells compared with HL-7702 cells. When treated with propranolol, both beta-1 and beta-2 receptors showed decreased expression on HepG2 and HepG2.2.15 cells. Therefore, the investigators concluded that, although beta-adrenergic receptors are more highly expressed in liver cancer cells than in normal liver cells, propranolol reduced their expression, inhibited proliferation, and induced apoptosis in liver cancer cells [97].

## 4. Conclusions

The use of propranolol in breast cancer patients may have promising effects based on its ability to reduce the proliferation of cancer cells and its potential ability to cause apoptosis in those cells. Studies have shown that nonselective BBs, for their action of both beta-1 and beta-2 adrenergic receptors, are able to reduce Ki-67 expression, an indication of decreased proliferation rate. Increased relapse-free survival and decreased rate metastasis and heart failure incidence were also found in the breast cancer patients evaluated.

Regarding pancreatic cancer, the use of BBs was associated with a reduction of cancer-specific mortality rate. However, no differences were found between patients treated with cardioselective and nonselective BBs.

The literature review performed in this study suggests that propranolol may be used as a complement for the treatment of several types of cancer, due to its ability to improve treatment outcomes, decreasing the proliferation rates of cancer cells, although this information needs to be clarified with further studies with well-defined target groups and placebo controlled conditions. A first approach may involve in vitro studies designed to provide responses to the potential mechanistic action of these drugs and their interaction with other pharmaceuticals, on different types of cancer cells.

Considering that the conventional chemotherapy affects cancer cells but may also attack healthy cells, smart nanocarrier-based drug delivery systems, consisting of a controlled drug release (e.g., by liposomes), which direct the drug release to the specific cancer site [98] are being developed. As phospholipid-based nanocarriers, liposomes improve pharmacokinetics, biodistribution, solubility, and stability and control release and site-specific delivery of the anti-cancer drugs, causing less side effects than conventional systems [99]. Therefore, nanocarriers could be studied in order to understand if their use decreases cardiac toxic effects related to anticancer chemotherapy.

## Figures and Tables

**Table 1:** Examples of frequently used beta blockers and antineoplastic agents.

Pharmaceutical	Class	Chemical Formula	Structure	CAS
Atenolol	BB-B1	C_14_H_22_N_2_O_3_	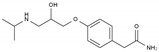	29122-68-7
Celiprolol	BB-B1	C_20_H_33_N_3_O_4_	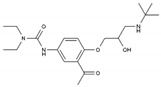	56980-93-9
Metoprolol	BB-B1	C_15_H_25_NO_3_	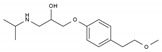	51384-51-1
Bisoprolol	BB-B1	C_18_H_31_NO_4_	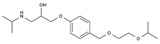	66722-44-9
Nebivolol	BB-B1	C_22_H_25_F_2_NO_4_	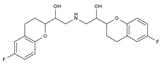	99200-09-6
Propranolol	BB-NS	C_16_H_21_NO_2_	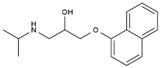	525-66-6
Sotalol	BB-NS	C_12_H_20_N_2_O_3_S	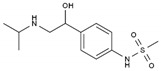	3930-20-9
Carvedilol	BB-NS	C_24_H_26_N_2_O_4_	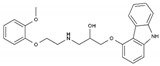	72956-09-3
Labetalol	BB-NS	C_19_H_24_N_2_O_3_	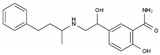	36894-69-6
Timolol	BB-NS	C_13_H_24_N_4_O_3_S	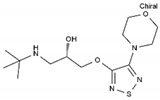	26839-75-8
Busulfan	AA-Alk	C_6_H_14_O_6_S_2_	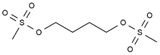	55-98-1
Carmustine	AA-Alk	C_5_H_9_Cl_2_N_3_O_2_	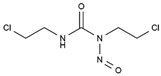	154-93-8
Cyclophosphamide	AA-Alk	C_7_H_15_Cl_2_N_2_O_2_P	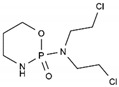	50-18-0
Estramustin	AA-Alk	C_23_H_31_Cl_2_NO_3_	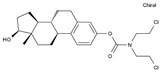	2998-57-4
Lomustine	AA-Alk	C_9_H_16_ClN_3_O_2_	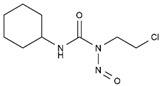	13010-47-4
Mesna	AA-Alk	C_2_H_5_NaO_3_S_2_	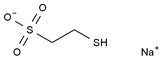	19767-45-4
Carboplatin	AA-AlkRel	C_6_H_12_N_2_O_4_Pt	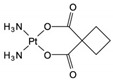	41575-94-4
Cisplatin	AA-AlkRel	Cl_2_H_6_N_2_Pt	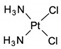	15663-27-1
Disulfiram	AA-AlkRel	C_10_H_20_N_2_S_4_ or ((C_2_H_5_)_2_NCS)_2_S_2_	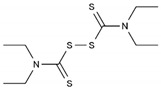	97-77-8
Procarbazine	AA-AlkRel	C_12_H_19_N_3_O	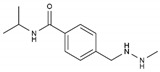	671-16-9
Fluorouracil	AA-AntMet	C_4_H_3_FN_2_O_2_	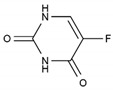	51-21-8
Methotrexate	AA-AntMet	C_20_H_22_N_8_O_5_	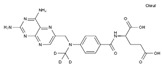	59-05-2
Tegafur	AA-AntMet	C_8_H_9_FN_2_O_3_	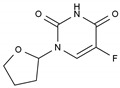	17902-23-7
Irinotecan	AA-Top-1Inh	C_33_H_38_N_4_O_6_	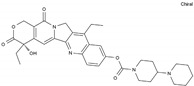	97682-44-5
Topotecan	AA-Top-1Inh	C_23_H_23_N_3_O_5_	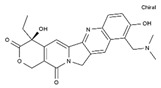	123948-87-8
Etoposide	AA-Top-2Inh	C_29_H_32_O_13_	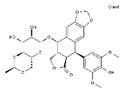	33419-42-0
Teniposide	AA-Top-2Inh	C_32_H_32_O_13_S	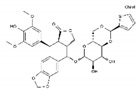	29767-20-2
Bleomycin	AA-DNAIntAg	C_55_H_84_N_17_O_21_S_3_^+^	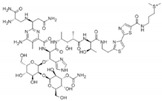	11056-06-7
Daunorubicin	AA-DNAIntAg	C_27_H_29_NO_10_	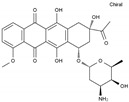	20830-81-3
Doxorubicin	AA-DNAIntAg	C_27_H_29_NO_11_	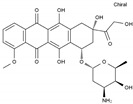	23214-92-8
Epirubicin	AA-DNAIntAg	C_27_H_29_NO_11_	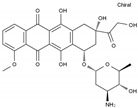	56420-45-2
Idarubicin	AA-DNAIntAg	C_26_H_27_NO_9_	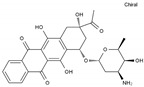	58957-92-9
Docetaxel	AA-IntTub	C_43_H_53_NO_14_	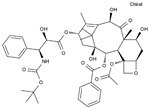	114977-28-5
Paclitaxel	AA-IntTub	C_47_H_51_NO_14_	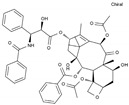	33069-62-4
Vinblastine	AA-IntTub	C_46_H_58_N_4_O_9_	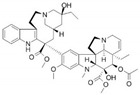	865-21-4
Vincristine	AA-IntTub	C_46_H_56_N_4_O_10_	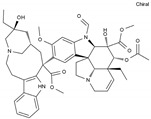	57-22-7
Imatinib	AA-TyrKinInh	C_29_H_31_N_7_O	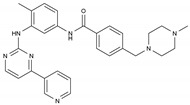	152459-95-5
Lapatinib	AA-TyrKinInh	C_29_H_26_ClFN_4_O_4_S	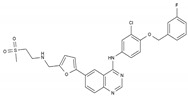	231277-92-2
Amsacrine	AA-Other	C_21_H_19_N_3_O_3_S	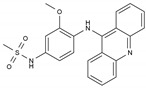	51264-14-3
Hydroxyurea	AA-Other	CH_4_N_2_O_2_	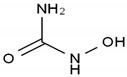	127-07-1
Pentostatin	AA-Other	C_11_H_16_N_4_O_4_	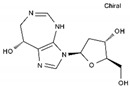	53910-25-1

BB—Beta Blocker; B1—Beta 1 selective; NS—Non-Selective; AA—Antineoplastic Agent; Alk—Alkylating, AlkRel—Alkylating Related; AntMet—Antimetabolite; Top-1Inh—Topoisomerase-1 inhibitor; Top-2Inh—Topoisomerase-2 inhibitor; DNAIntAg—DNA-intercalating agents; IntTub—Interfere with tubulin; TyrKinInh—Tyrosine-kinase inhibitors.
